# Collective memory for political leaders in a collaborative government system: Evidence for generation-specific reminiscence effects

**DOI:** 10.3758/s13421-020-01076-8

**Published:** 2020-08-05

**Authors:** Beat Meier

**Affiliations:** Institute of Psychology, University of Bern, Fabrikstr. 8, 3000 Bern, Switzerland

**Keywords:** Recall, Autobiographical memory, Memory

## Abstract

Collective memory is shared by a group and is part of that group’s identity. Memory for political leaders is a prototypical case of collective memory. The present study investigated collective memory for Swiss federal councilors in order to test the trajectory of collective memory across four different generations (i.e., Millennials, Generation X, Baby-Boomers, and Silents) in a collaborative government system. In contrast to a presidential system, Switzerland is governed by seven equal councilors who share power and responsibilities. Thus, the individual member of the government is less important, and the number of councilors is larger compared to a presidential system, which may influence collective memory. The results revealed a recency effect as well as a generation-specific reminiscence effect, but no primacy effect as reported for presidential systems. These results indicate that the contribution of semantic memory and autobiographic memory to the trajectory of collective memory vary across government systems. Specifically, for a collaborative government system, autobiographic memory has a stronger contribution to the trajectory of collective memory.

## Introduction

Collective memory is a form of memory that is shared by a group and is part of that group’s identity. Although the concept was introduced nearly a century ago in order to emphasize that individual memories are to be understood in a group context in which people anchor their identity (Halbwachs, 1925), the phenomenon has only recently become a focus from a psychology-of-memory perspective (Hirst, Yamashiro, & Coman, 2018; Pennebaker, Páez, & Rime, 1997; Roediger & Abel, 2015). In a seminal study, Roediger and DeSoto (2014) tested the collective memory for US presidents across three different generations (Baby Boomers, Generation X, and Millennials). Besides a recency effect, they also found a primacy effect, that is, better memory for the first US presidents (cf. Roediger & Crowder, 1976). A similar result was reported for Canadian prime ministers (Neath & Saint-Aubin, 2011) and for Chinese leaders (Fu, Xue, DeSoto, & Yuan, 2016).

The present study was designed to test whether these findings from single-leader systems would replicate for collective memory of Swiss federal councilors. In contrast to the USA, Canada, and China, Switzerland is governed by seven equal councilors (“Bundesräte” in German), thus, there is also a much larger number of (former) leaders. In fact, since the establishment of the modern Swiss federal state in 1848, more than a hundred councilors have ruled Switzerland. As a consequence, single councilors may be less important and memory for the first few leaders may be less pronounced than in presidential systems. Moreover, based on the findings from list-learning studies (Murdock, 1962; Ward, 2002), one would assume that the higher number of names in the Swiss system would lead to a higher number but a lower proportion of names remembered compared to single-leader systems. In Switzerland, the names of all the individual councilors are not systematically learned at school and there is no particular emphasis on the founding members of the modern Swiss federal state (Grube, 2002). Thus, the existence of a collective primacy effect as reported for US presidents seems less likely as this effect is rather due to the emphasis in history education than due to a memory of the original event. The present study extended the range of different age cohorts by including the generation of Silents (older adults born between 1924 and 1948), besides Millennials, Generation X, and Baby Boomers. Moreover, based on the literature on autobiographical memory, the hypothesis that a reminiscence effect may occur for collective memory, in different decades and for each different generation, was tested.

Given the fact that a marked increase of memories from the period between the ages of 15 and 30 years can be observed when people are probed for autobiographical memories, as well as for public events (Janssen & Murre, 2008; Janssen, Murre, & Meeter, 2008; Rubin, Rahhal, & Poon, 1998; Rubin, Wetzler, & Nebes, 1986; Schuman, Akiyama, & Knauper, 1998; Schuman, Belli, & Bischoping, 1997; Schuman & Corning, 2012, 2014; Schuman & Scott, 1989; Zaromb, Butler, Agarwal, & Roediger, 2014), it seems quite surprising that no such generation-specific reminiscence effect was observed in any of the previous studies on collective memory for political leaders in single-leader systems. One reason for the lack of such generation-specific reminiscence effects may be that in single-leader systems, political leaders are very well learned or that the length of the list of leaders is limited, which prevents the expression of an autobiographical memory bump.

From the perspective of cognitive development, this period is the time in which most cognitive functions such as speed of performance and memory abilities peak. It is a time with many important events and decisions, a time of identity formation in which individuals typically also get involved in politics (e.g., because they obtain the right to vote). Therefore, it seems likely that for each individual, memory for those councilors who were elected during this period should also be enhanced. Compared to a presidential system with only one person in charge, the larger number of leaders in the Swiss political system also presents more opportunities for variability in memory performance, and, thus, it may be easier to detect a reminiscence effect.

Better collective memory for more recent events has been found quite generally across different subjects such as the names of former political leaders, aircraft crashes, citation of academic articles and patents, songs, movies, and biographies (Candia, Jara-Figueroa, Rodriguez-Sickert, Barabasi, & Hidalgo, 2019; Fu et al., 2016; Garcia-Gavilanes, Mollgaard, Tsvetkova, & Yasseri, 2017; Roediger & DeSoto, 2014; Spivack, Philibotte, Spilka, Passman, & Wallisch, 2019). Therefore, a recency effect was expected in the present study.

Thus, this study tested whether the pattern that was found for US presidents (Roediger & DeSoto, 2014) and to some degree also for Canadian prime ministers (Neath & Saint-Aubin, 2011), and Chinese leaders (Fu et al., 2016), namely a primacy effect and a recency effect would also occur for Swiss federal councilors. Moreover, inspired by research on autobiographical memory, the hypothesis that there should be generation-specific reminiscence effects for collective memory of Swiss federal councilors was tested.

## Method

### Participants

A total of 408 participants were recruited for the study. Data collection was administered as part of a research methods class and took place in October/November 2017 and October/November 2018. Selection criteria were that the participants were educated in the Swiss school system, that they lived in Switzerland at least since 7 years of age, and that they belonged to one of four generations, Millennials (born 1989–2000), Generation X (born 1969–1988), Baby Boomers (born 1949–1968), or Silents (born 1924–1948). Participants were recruited among family, friends, colleagues, and members of various organizations (e.g., sports clubs, gym, etc.). The resulting sample consisted of 103 Millennials (mean age = 23.7 years, 55% female), 88 people from Generation X (mean age = 38.0 years, 56% female), 118 Baby Boomers (mean age = 56.2 years, 53% female), and 99 Silents (mean age = 78.0 years, 48% female). The study was approved by the ethics committee of the Faculty of Human Sciences of the University of Bern and all methods were performed in accordance with the relevant guidelines and regulations.

### Materials and procedure

For recruitment, the participants were informed that the study was on memory, that the goal was to compare how well people from different age groups can remember the names of specific people, and that it would take less than 10 min to participate. When they agreed to participate, they were asked whether they went through the Swiss school system, and if the response was affirmative, they were invited to take part in the study. Participants were given a pen and a ruled sheet of paper with the instructions to recall and write down the names of all Swiss federal councilors that they could remember, in any order. They were given 3 min to accomplish this task. Pilot testing had shown that this amount of time was sufficient and that giving more time was not helpful for participants.

At the time of the study, Switzerland had a total of 117 present or former councilors. Five of them were elected in the 2010 decade, eight were elected in the decade between 2000 and 2009, five between 1990 and 1999, ten between 1980 and 1989, seven between 1970 and 1979, six between 1960 and 1969, 12 between 1950 and 1959, seven between 1940 and 1949, etc. The full list of Swiss federal councilors together with their election date can be found on Federal Council’s Webpage (https://www.admin.ch/gov/en/start/federal-council/history-of-the-federal-council/federal-council-elections-since-1848/alle-bundesraete-liste.html).

### Design and analysis

For data analysis, the answers of the participants were transcribed and the councilors were ordered according to their election date. Based on the election date, they were assigned to a certain decade that was subsequently used for analysis (see Janssen, Gralak, & Murre, 2011, for a similar approach). Once elected, Swiss federal councilors are typically confirmed in their position every 4 years and are typically part of the government until they resign. On average a councilor remains in office for 10 years. As the term in office is different for each individual councilor, the election date was used as a reference point to assign a councilor to a certain decade. As the number of new elections per decade varies across time, a proportion score rather than the absolute number of remembered names of councilors was used for analysis. For each decade, the sum of recalled councilors was divided by the total number of councilors in that period, and the resulting probability was used as the dependent variable. As it turned out that the number of recalled councilors before the 1940 decade was very low, the periods between 1900 and 1939 and between 1848 and 1899 were summarized, resulting in a total of ten periods, which for the purpose of analysis are labelled decades.

In Table 1, the number of councilors elected per decade and their time in office is listed (except for the 2010–2018 decade because only two out of the seven elected in this period have already resigned). To control whether time in office differed across decades and thus may have had an influence on memory performance, an analysis of variance (ANOVA) was conducted. This gave *F* (6,47) = .839, *p* = .54, η^2^ = .097, suggesting that time in office does not differ across decades.

**Table 1:** Number of councilors elected in each period and average time in office

Decade	Councilors	Days in office	SD
2000	7	2,964	926
1990	5	3,561	1,440
1980	10	3,451	1,806
1970	7	3,510	1,091
1960	6	3,482	960
1950	12	2,662	1,422
1940	7	3,906	1,303
1900–1939	21	4,034	2,694
Before 1900	36	4,418	2,832

Originally, to test the trajectory of collective memory for Swiss federal councilors, the design was planned as two-factorial with the between-subjects factor generation (Millennials, Generation X, Baby Boomers, and Silents) and the within-subject factor decade (2010–2018, 2000–2009, 1990–1999, 1980–1989, 1979–1979, 1960–1969, 1950–1959, 1940–1949, 1900–1939, 1848–1899). As screening of the data revealed that the assumption of normality was violated, non-parametric tests were used. In order to test for a recency effect, memory for the councilors from the most recent decades (2010–2018, 2000–2009, 1990–1999) were compared with a related-samples Wilcoxon signed-ranked test, separately, and for each generation (Millennials, Generation X, Baby Boomers, and Silents). In order to test for a primacy effect, a similar analysis was planned for the first periods. However, as it turned out, memory was at floor level for the councilors from the 1848–1899 period, and thus no meaningful primacy effect analysis across generations was possible. In order to test for a reminiscence effect, non-parametric Kruskal-Wallis tests were run with the generation factor, separately for each relevant decade, followed by pairwise comparisons. As the reminiscence effect of the oldest generation, the Silents, cannot occur before the 1940 decade, the range of these analyses was restricted accordingly. Asymptotic significances are reported (two-sided) and significance levels for post hoc tests are adjusted by the Bonferroni correction for multiple tests. *R* was calculated as effect size for non-parametric tests, with *r* = .10, *r* =. 30 and *r* =.50 indicating small, medium, and large effects, respectively. For all statistical analyses, an alpha level of .05 was used.

## Results

On average, the number of remembered councilors was *M* = 10.04 (*SD* = 4.738). Across generations, mean memory performance was 7.72 (*SD* = 3.321) for Millennials, 11.25 (*SD* = 4.698) for Generation X, 11.20 (*SD* = 4.919) for Baby Boomers, and 9.98 (*SD* = 4.965) for Silents. An ANOVA revealed a significant group difference, *F* (3, 408) = 13.707, *p* < .001, η^2^ = .092. Post hoc t-tests showed that this effect was mainly due to the Millennials who remembered fewer councilors than all the other groups (all *p*s < .001, *d*s > .537). There was also a marginally significant difference between Generation X and Silents (*p* = .056, *d* = .262) and between Baby Boomers and Silents (*p* = .048, *d* = .247), while the difference between Generation X and Baby Boomers was not significant (*p* = .994; *d* = .01).

**Fig. 1:**
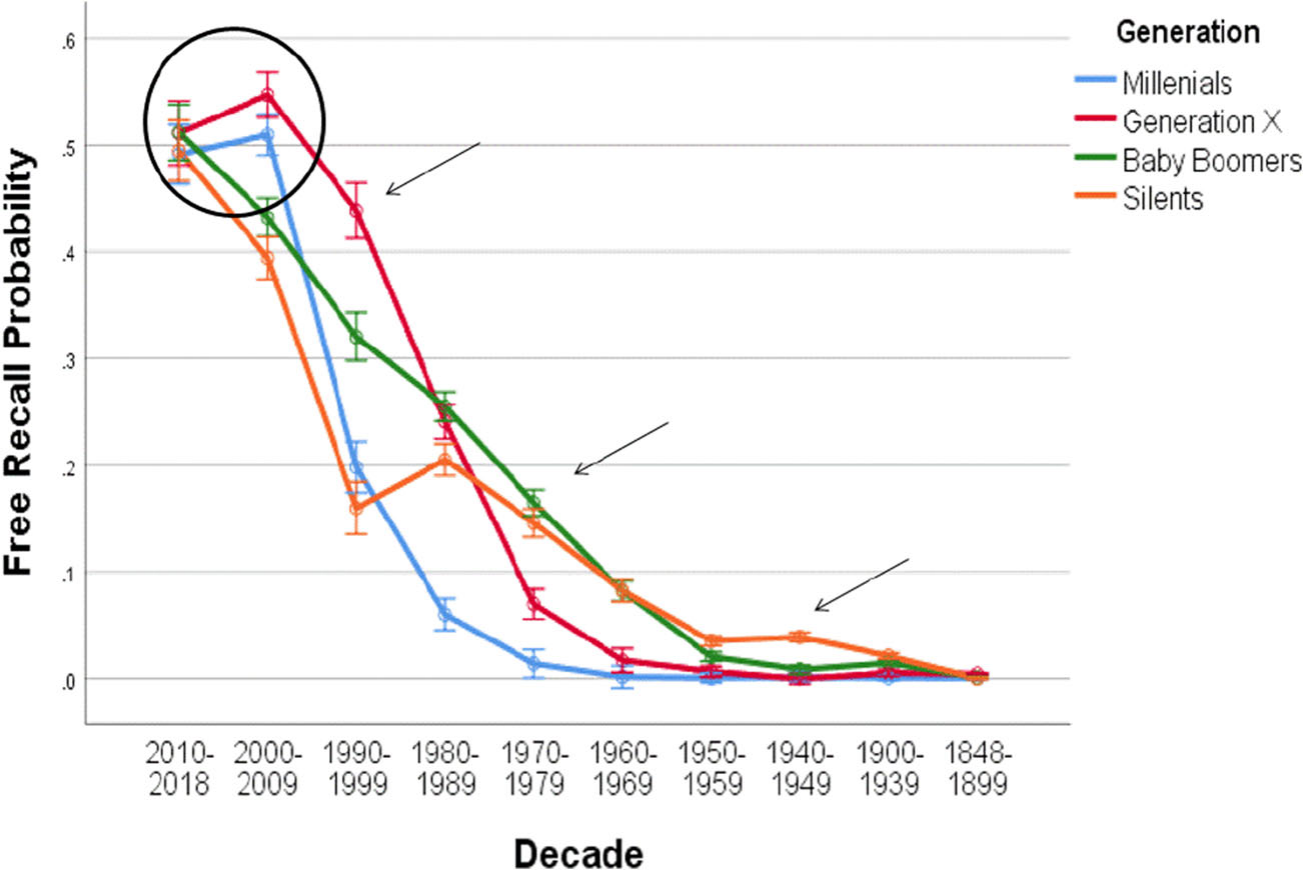
Free recall probability. Free recall trajectory of Swiss federal councilors elected in each decade since the establishment of the modern Swiss federal state for four different generations (Millennials, Generation X, Baby Boomers, and Silents) shows a recency effect (circle) and generation-specific reminiscence effects (arrows). Error bars indicate standard errors

The trajectory of collective memory for Swiss federal councilors in terms of recall probability for each different generation is depicted in Fig. 1. Overall, the pattern of recall probability indicates a similar trajectory as is typically found for explicit episodic memory, that is, better memory for more recent compared to more distant information. The results do not show an indication of a primacy effect, rather performance was at floor level for councilors elected between 1848 and 1899, *M* = .001, *SD* = .0094. As can be seen in Fig. 1, although also very low, probability of recall for councilors elected between 1900 and 1939 was somewhat higher (*M* = .011, *SD* = .0277). Thus, if anything, there is a (slightly) better memory for councilors from the latter time period rather than any indication of a primacy effect. A related-samples ranked Wilcoxon signed-rank test showed that this difference is significant, *Z* = 7.014, p < .001, *r* = .34. However, the results seem to suggest reminiscence effects, inherently at separate periods for each generation. Therefore, only the recency effect and the reminiscence effect were followed up statistically across generations.

To test for a recency effect, memory performance for councilors from the last two decades (2010–2018, 2000–2009) was initially compared in the whole sample. A related-samples Wilcoxon signed-rank test showed better performance for the 2010–2018 decade than for the 2000–2009 decade, *Z* = -2.376, *p* = 0.017, *r* = .12, indicating a recency effect. However, as Fig. 1 suggests, this pattern is only evident for Baby Boomers and Silents, not for Millennials and Generation X, who seem to have a prolonged recency effect that included the two recent decades, so separate analyses were calculated for each group. These comparisons gave significant effects for Baby Boomers and Silents (*Z* = -3.381 and *Z* = -3.153, respectively, *p*s < .005, *r*s >.31), but not for Millennials and Generation X (*Z* = .782, *p* = .434, *r* =.078 and *Z* = 1.146, *p* = .252, *r* = .12, respectively). As it is possible that the recency effect was prolonged by a reminiscence effect in the two younger groups, further comparisons for councilors elected in the 2000 decade and the 1990 decade were calculated. These showed a difference for all generations (all *Z*s < -3.35, *p*s < .001, *r*s > .30). This indicates that the recency effect of the two younger cohorts lasted longer, probably due to an additional boost provoked by a reminiscence effect (cf. Janssen et al., 2011).

To test for potential reminiscence effects more directly, memory for councilors for each decade was analyzed separately. For the 2010 decade, an independent-samples Kruskal-Wallis test gave no difference between groups, χ2 (3, N = 408) = .496, *p* = .920, ε^2^ = .001, indicating that for the most recent decade collective memory was similar for each generation. In contrast, for the 2000 decade, the analysis gave a significant effect, χ2 (3, N = 408) = 31.082, *p* < .001, ε^2^ = .08. The results of the Bonferroni post hoc tests revealed that memory performance was better for Millennials and Generation X compared to Baby Boomers and Silents (*p*s < .05, *r*s > .18), while the comparisons between Millennials and Generation X, and Baby Boomers and Silents, respectively, were not significant (*r*s < .10). The relative advantage for both younger generations thus supports the hypothesis that the prolonged recency effect may be due to a reminiscence effect. For the 1990 decade, the analysis was also significant, χ2 (3, N = 408) = 62.091, *p* < .001, ε^2^ = .15. Here, the post hoc tests revealed that Generation X outperformed all other generations (*p*s < .05, *r*s > .19), providing further evidence for the presence of a reminiscence effect. Baby Boomers showed better performance than both Millennials and Silents (*p*s < .001, *r*s > .24), while the latter two groups did not differ (*r* = .07). For the 1980 decade, the analysis was also significant, χ2 (3, N = 408) = 107.291, *p* < .001, ε^2^ = .26. Here, Millennials showed lower memory compared to all other generations (*p*s < .001, *r*s >.48), while the other comparisons gave no significant effects (*p*s > .05, *r*s < .15). For the 1970 decade, the analysis was also significant, χ2 (3, N = 408) = 85.634, *p* < .001, ε^2^ = .21. Baby Boomers and Silents outperformed the two younger generations (*p*s < .005, *r*s >.27), and Generation X performed better still than Millennials (*p* = .004, *r* = .25). For the 1960 decade, the analysis was also significant, χ2 (3, N = 408) = 55.933, *p* < .001, ε^2^ = .14. Again, post hoc tests showed that Baby Boomers and Silents outperformed the two younger generations (*p*s < .001, *r*s > .28), but no other comparison was significant (*r*s < .10). For the 1950 decade, the analysis was also significant, χ2 (3, N = 408) = 35.644, *p* < .001, ε^2^ = .09. Silents outperformed both Millennials and Generation X (*p*s < .001, *r*s > .30), and Baby Boomers were better than Millennials (*p* = .002, *r* = .24). No other effect was significant (all *p*s >.13, *r*s < .16). For the 1940 decade, the analysis was also significant, χ2 (3, N = 408) = 55.933, *p* < .001. Silents outperformed all the younger generations (*p*s < .001, *r*s > .31). All other comparisons were not significant (*r*s < .11). Despite this robust statistical effect, it is important to note that floor effects may have exaggerated the differences. Nevertheless, the reminiscence effect for the Silents is consistent with the hypothesis.

Thus, the overall pattern of these analyses showed a shifting relative memory advantage across generations and decades, suggesting that a reminiscence effect was present for Generation X for councilors elected in the decades 2000–2009 and 1990–1999, for Baby Boomers for councilors elected in the decades 1980–1989, 1970–1979, and 1960–1969, and for Silents for councilors elected in the decades 1960–1969, 1959–1959, and 1940–1949.^1^

## Discussion

The present study was designed to test whether the findings from the seminal study by Roediger and DeSoto (2014) in which collective memory for US presidents revealed both a primacy and a recency effect across several generations (i.e., Millennials, Generation X, and Baby Boomers) would replicate for collective memory of Swiss federal councilors. It extended the range of different age cohorts by including the generation of Silents (older adults born between 1924 and 1948), besides Millennials, Generation X, and Baby Boomers. Moreover, based on the literature on autobiographical memory, the hypothesis that a reminiscence effect may occur for collective memory, in different decades and for each different generation, was tested.

In line with Roediger and DeSoto and other previous studies on collective memory for a nation’s leader (Fu et al., 2016; Neath & Saint-Aubin, 2011), the results of the present study also revealed a recency effect, that is, better memory for more recent councilors. For the two older generations, a steady decline in recall probability was evident across the most recent decades. For the two younger generations, the recency effect was more pronounced and included the two most recent decades before a memory decline was evident. The bump that was observed for the 2000–2009 decade for these two groups is likely to include a reminiscence effect, and the decade-wise analysis has supported this interpretation. There was a steady shift in the relative memory advantage that was specific for the period in which each generation was between the ages of about 15 and 30 years, that is, the time in which individuals typically get involved in politics. Specifically, Generation X showed a reminiscence effect for the 1990–1999 decade, during which they were between the ages of 12 and 21 years. Baby Boomers showed a reminiscence effect for the 1970–1979 decade, during which they were also between the ages of 12 and 21 years. Finally, Silents showed a reminiscence effect for the 1950–1959 and the 1940–1949 decades, respectively, during which they were between the ages of 8 and 23 years. Notably, these age ranges rather underestimate the exact age at which the reminiscence effect occurred because for each councilor the data for the time at which he was elected was used as the timing criterion. As each councilor remains in office for 10 years on average, the contribution to the autobiographical reminiscence bump may vary within individuals.

The results of the present study did not show any indication of a primacy effect. In fact, memory probability for councilors from the early days of the modern Swiss federal state between 1848 and 1899 was at floor level. The lack of a primacy effect differs from the findings in presidential/single-leader political systems, in which the first leader was typically also remembered considerably well (DeSoto & Roediger, 2019). It is likely that this result is related to the fact that names of the founding councilors are not learned at school.

Thus, compared to studies of presidential or single-leader systems (DeSoto & Roediger, 2019), there are both similarities and differences. The similarity is the consistent recency effect, that is, better performance for more recent politicians. Roediger and DeSoto (2014) suggested that the recency effect indicated that particpants can retrieve those presidents relatively well, who held office during or just before their lifetimes. In the present study the recency effect rather indicates memory for the actual councilors who are (still) in office. Moreover, in the present study, the recency effect was prolonged in the two younger generations. This effect may be due to autobiographical memory, which typically shows a bump for events that occur in young adulthood. There was also a consistent pattern of generation-specific reminiscence bumps.

Finally, while studies on single-leader systems all reported a substantial primacy effect for the first leaders, this effect did not occur for Swiss federal councilors. In order to integrate the differences in the results of these studies, it is important to acknowledge that they are likely due to the differences of number of councilors and accordingly differences in the importance of each individual. While in the presidential systems the national leader has stronger power and media presence, in the Swiss system each councilor is part of a collaborative authority with a more restricted range of power and shared media presence. Thus, both the number of leaders to remember and the relative importance of each leader may affect collective memory. Moreover, after their time in office, leaders of single-leader systems are represented differently in a historical context. They are attributed with specific accomplishments and later receive attention indirectly through books in history lessons at school. This is much less the case for a collective memory system, in which from a perspective of memory encoding there are fewer learning opportunities both during time in office as well as afterwards.

Another important difference relates to the source of collective memory. First, collective memory may be rooted in semantic memory, that is, information that has been acquired without specific reference to the time and place it was acquired. Most knowledge that we learn in school can be considered semantic knowledge. Therefore, memory for the names of political leaders such as the first US president is likely supported by this kind of memory. Second, collective memory can be based on episodic, autobiographic memory, that is, experiences that are connected to a specific time and place (Tulving, 2002). As a result, the contribution of semantic memory and autobiographic memory to collective memory may vary across study materials and situations.

Specifically, the contribution of autobiographical memory may be less pronounced in situations in which fewer retrieval items are present, such as the number of US presidents (on average about two per decade) versus the number of Swiss federal councilors (on average about ten per decade) and in a situation in which the retrieval items are less distinctive such as in a collaborative system as in Switzerland versus a presidential system as in the USA. Accordingly, in the present study, the contribution from autobiographical memory may have been much more pronounced than the contribution from semantic memory, thus revealing generation-specific reminiscence bumps. In contrast, the contribution of semantic memory may have been less pronounced, which can explain why there was no primacy effect.

Along another line of thought, the Swiss may be modest people who do not want to overemphasize single individuals. This would complement the fact that in a survey on national narcissism, Swiss scored lower than all other participating countries (Zaromb et al., 2018).

Last, but not least, the recall measure may have a particular effect on serial position effects. For example, serial position effects have been found in a study on fight song lyrics when memory for order was prompted but not when free recall was prompted (Overstreet & Healy, 2011). However, other research indicates that the particular memory measure cannot explain the different results. For example, the study on Canadian prime ministers also assessed free recall memory, and both primacy and recency effects were found (Neath & Saint-Aubin, 2011). Similarly, the larger number of councilors (compared, e.g., to the number of US presidents (i.e., list-length effects)) cannot explain the lack of a primacy effect because even with very long lists, a primacy effect is typically found. Thus, the lack of a primacy effect is rather related to a lack of learning the first councilors at all. As a consequence, one would also not expect that a “primacy effect” would occur with a recognition memory test (i.e., it is a problem of availability, not accessibility).

The present study complements findings from autobiographical memory research where a reminiscence effect has been found with various procedures and populations (Janssen & Murre, 2008; Rubin et al., 1986). The finding of a generation-specific reminiscence effect for political leaders also complements similar findings outside the autobiographical memory domain such as memory distributions and ratings of importance of public events (Janssen et al., 2008; Schuman & Corning, 2012).

Moreover, the present study provides an explanation for why no such reminiscence effects have been found in previous studies on memory for former political leaders (Fu et al., 2016; Neath & Saint-Aubin, 2011; Roediger & Crowder, 1976; Roediger & DeSoto, 2014). In fact, all of these studies addressed single-leader political systems. Inherently, single-leader systems result in fewer leaders and greater emphasis on each single person, which may have covered the expression of an autobiographical reminiscence bump.

To summarize, the present study indicates that while a recency effect seems to be a general feature of collective memory for political leaders, the existence of a reminiscence bump and a primacy effect differ across government systems. The present study indicates that in a collaborative government system collective reminiscence bumps may be revealed.

## Footnotes

1 I would like to thank an anonymous reviewer for suggesting to obtain an “objective” measure of the amount of information available of each Swiss federal councilors via a Google search and to test how it relates to memory performance. Following the approach of Neath and Saint-Aubin (2011), I ran a Google search (searching for: “name” councilor, i.e. “Name” Bundesrat, in German) for each councilor The particular values were collected between May 22 and May 28, 2020. Overall, there was a strong correlation between Google Hits and memory performance (r = .70), but notably the correlation between year of election and memory performance was even higher (r =. 81). This pattern remained when only the councilors elected from 1940 were taken into account with r = .67 and r = .85, respectively. The results suggest that overall the objective amount of information available of each Swiss federal councilors is strongly related to individual memory performance.
